# Supplementary material for: Construction of relatedness matrices using genotyping-by-sequencing data
Source: BMC Genomics. 2015 Dec 9;16:1047. doi: 10.1186/s12864-015-2252-3 (PMC4675043; doi:10.1186/s12864-015-2252-3)
Supplement: Additional file 1: — Further details of calculations of expected values of relevant functions of genotype scores. (PDF 122 kb) [file 12864_2015_2252_MOESM1_ESM.pdf]

Additional file 1. Further details of calculations of expected values of relevant functions of genotype scores.

$$\begin{aligned}
E(x_i) &= 2P(\mathrm{AA}_i^*) + P(\mathrm{AB}_i^*)\\
&= 2p^2 + 2p(1-p)F_i + 4p(1-p)(1-F_i)K_i + 2p(1-p)(1-F_i)(1-2K_i)\\
&= 2p^2 + 2p(1-p)[F_i + 2(1-F_i)K_i + (1-F_i)(1-2K_i)]\\
&= 2p^2 + 2p(1-p)[F_i + 2K_i - 2F_iK_i + 1 - F_i - 2K_i + 2F_iK_i]\\
&= 2p^2 + 2p(1-p)\\
&= 2p(p+1-p)\\
&= 2p\\
E(x_i^2) &= 4P(\mathrm{AA}_i^*) + P(\mathrm{AB}_i^*)\\
&= 4p^2 + 4p(1-p)F_i + 8p(1-p)(1-F_i)K_i + 2p(1-p)(1-F_i)(1-2K_i)\\
&= 4p^2 + 2p(1-p)[2F_i + 4(1-F_i)K_i + (1-F_i)(1-2K_i)]\\
&= 4p^2 + 2p(1-p)[2F_i + 4K_i - 4F_iK_i + 1 - F_i - 2K_i + 2F_iK_i]\\
&= 4p^2 + 2p(1-p)(1 + F_i + 2K_i - 2F_iK_i)\\
E(x_ix_i') &= 4P(\mathrm{AA}_i^*, \mathrm{AA}_{i'}^*) + 2P(\mathrm{AA}_i^*, \mathrm{AB}_{i'}^*) + 2P(\mathrm{AB}_i^*, \mathrm{AA}_{i'}^*) + P(\mathrm{AB}_i^*, \mathrm{AB}_{i'}^*)\\
&= 4P(\mathrm{AA}_i, \mathrm{AA}_{i'}) + 2P(\mathrm{AA}_i, \mathrm{AB}_{i'})(2K_{i'} + 1 - 2K_{i'}) + 2P(\mathrm{AB}_i, \mathrm{AB}_{i'})\\
&\qquad + P(\mathrm{AB}_i, \mathrm{AB}_{i'})(4K_iK_{i'} + 2K_i(1-2K_{i'}) + 2K_{i'}(1-2K_i) + (1-2K_i)(1\\
&\qquad - 2K_{i'}))\\
&= 4P(\mathrm{AA}_i, \mathrm{AA}_{i'}) + 4P(\mathrm{AA}_i, \mathrm{AB}_{i'}) + P(\mathrm{AB}_i, \mathrm{AB}_{i'})\\
&= 4p(1-p)(1-6p+6p^2)\delta + 12p^2(1-p)^2\Delta + 16p^2(1-p)(1-2p)\gamma\\
&\qquad + 24p^3(1-p)\theta + 4p^4 + 8p(1-p)[-(1-6p+6p^2)\delta - 3p(1-p)\Delta + (1\\
&\qquad - 2p)(1-4p)\gamma + 3p(1-2p)\theta + p^2] + 4p(1-p)[(1-6p+6p^2)\delta\\
&\qquad + 3p(1-p)\Delta - 2(1-2p)^2\gamma + (1-6p+6p^2)\theta + p(1-p)]\\
&= 4p(1-p)(1-6p+6p^2)\delta + 12p^2(1-p)^2\Delta + 16p^2(1-p)(1-2p)\gamma\\
&\qquad + 24p^3(1-p)\theta + 4p^4 + [-8p(1-p)(1-6p+6p^2)\delta - 24p^2(1-p)^2\Delta\\
&\qquad + 8p(1-p)(1-2p)(1-4p)\gamma + 24p^2(1-p)(1-2p)\theta + 8(1-p)p^3]\\
&\qquad + [4p(1-p)(1-6p+6p^2)\delta + 12p^2(1-p)^2\Delta - 8p(1-p)(1-2p)^2\gamma\\
&\qquad + 4p(1-p)(1-6p+6p^2)\theta + 4p^2(1-p)^2]\\
&= 4[1-2+1]p(1-p)(1-6p+6p^2)\delta + 12[1-2+1]p^2(1-p)^2\Delta + 8[2p\\
&\qquad + (1-4p) - (1-2p)]p(1-p)(1-2p)\gamma + 4[6p^2 + 6p(1-2p) + (1\\
&\qquad - 6p + 6p^2)]p(1-p)\theta + 4(p^2 + 2(1-p)p + (1-p)^2)p^2\\
&= 4[6p^2 - 12p^2 + 1 + 6p^2]p(1-p)\theta + 4(p^2 + 2p - 2p^2 + 1 - 2p + p^2)p^2\\
&= 4p(1-p)\theta + 4p^2\\
E(S_{ii}) &= E(x_i^2) - 4pE(x_i) + 4p^2\\
&= 4p^2 + 2p(1-p)(1 + F_i + 2K_i - 2F_iK_i) - 8p^2 + 4p^2\\
&= 2p(1-p)(1 + F_i + 2K_i - 2F_iK_i)\\
E(S_{ii'}) &= E(x_ix_{i'}) - 4pE(x_i) + 4p^2\\
&= E(x_ix_{i'}) - 4p^2\\
&= 4p(1-p)\theta_{ii'}
\end{aligned}$$
