# Supplementary figures and images for: Construction of relatedness matrices using genotyping-by-sequencing data
Source: BMC Genomics. 2015 Dec 9;16:1047. doi: 10.1186/s12864-015-2252-3 (PMC4675043; doi:10.1186/s12864-015-2252-3)

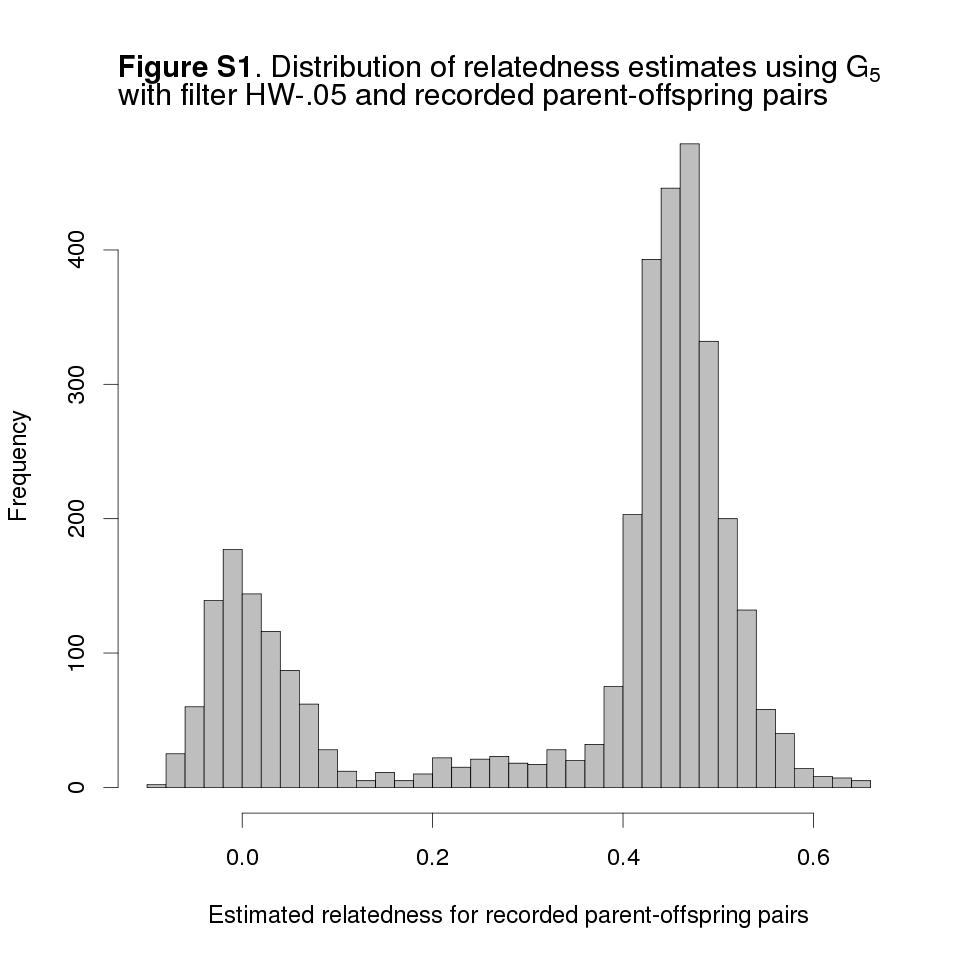

Supplement: Additional file 3: Figure S1. — Distribution of relatedness estimates using filter HW-0.05 and recorded parent-offspring pairs. (PNG 19 kb) [file 12864_2015_2252_MOESM3_ESM.png]

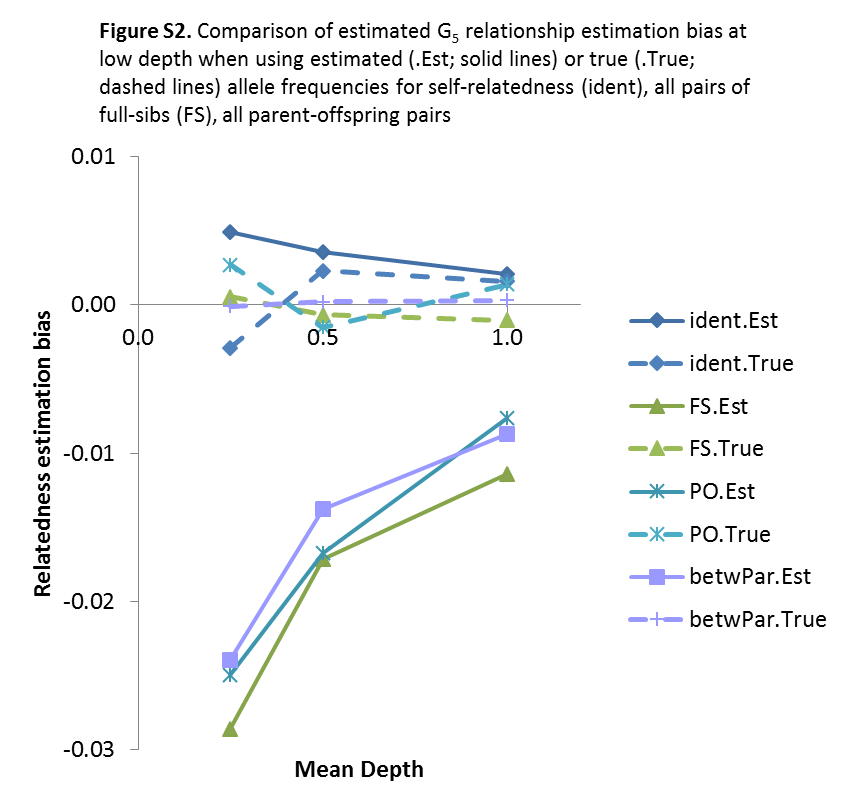

Supplement: Additional file 4: Figure S2. — Comparison of estimated relationship estimation bias at low depth when using estimated or true allele frequencies. (PNG 61 kb) [file 12864_2015_2252_MOESM4_ESM.png]
